# Balloon aortic valvuloplasty for critical aortic stenosis in neonates and infants: early outcomes from a single-center cohort

**DOI:** 10.1186/s12872-026-06170-4

**Published:** 2026-07-04

**Authors:** Elnur Imanov, Esmira M. Nasibova, Esmira M. Nasibova

**Affiliations:** ¹Azerbaijan Medical University, Baku, Azerbaijan

**Keywords:** Balloon aortic valvuloplasty, Critical aortic stenosis, Neonates, Infants, Congenital heart disease, Pediatric cardiology

## Abstract

**Background:**

Critical aortic valve stenosis in neonates and infants is a life-threatening congenital heart disease characterized by severe left ventricular outflow tract obstruction and compromised systemic perfusion. Balloon aortic valvuloplasty (BAV) has become an established initial treatment option; however, data on early outcomes in this vulnerable population remain relatively limited.

**Methods:**

This retrospective single-center study included 30 neonates and infants (≤ 12 months) with critical aortic valve stenosis who underwent balloon aortic valvuloplasty between 2016 and 2024. Clinical, echocardiographic, and hemodynamic parameters were analyzed before and after the procedure. Early procedural success, complications, and short-term outcomes were assessed.

**Results:**

Balloon aortic valvuloplasty resulted in a significant immediate reduction in transaortic pressure gradients (*p* < 0.05), indicating effective relief of left ventricular outflow tract obstruction. Left ventricular systolic function improved or remained stable in the majority of patients. New-onset or increased aortic regurgitation occurred in some cases, predominantly mild to moderate in severity, while severe regurgitation was uncommon. Early mortality was low, and most patients demonstrated favorable early clinical stabilization. Some patients required reintervention or surgical referral because of residual stenosis or progressive aortic regurgitation.

**Conclusions:**

Balloon aortic valvuloplasty is a safe and effective first-line treatment for critical aortic valve stenosis in neonates and infants, providing rapid hemodynamic improvement with acceptable early outcomes. The procedure represents an important therapeutic strategy in the initial management of this high-risk pediatric population.

## Introduction

Critical aortic valve stenosis in neonates and infants represents one of the most severe forms of left ventricular outflow tract obstruction encountered in pediatric cardiology. The condition is characterized by markedly elevated transaortic pressure gradients, impaired left ventricular systolic function, and compromised systemic perfusion, often resulting in duct-dependent circulation in the neonatal period. Without timely intervention, critical aortic stenosis may rapidly progress to cardiogenic shock, metabolic acidosis, and death.

Historically, surgical valvotomy was considered the primary therapeutic approach; however, surgery in neonates and young infants is associated with significant perioperative morbidity and mortality. Over the past decades, advances in interventional cardiology have led to the widespread adoption of balloon aortic valvuloplasty as an initial, less invasive treatment strategy. BAV offers the potential for rapid relief of obstruction, stabilization of systemic circulation, and postponement or avoidance of early surgical intervention.

Despite its established role, balloon aortic valvuloplasty in early infancy remains technically challenging. The risk of procedure-related complications, particularly the development of aortic regurgitation, as well as the need for reintervention, continues to raise clinical concerns. Furthermore, outcome data from single-center pediatric cohorts remain valuable, as patient characteristics, valve morphology, and institutional experience may significantly influence results.

The present study aims to evaluate the early clinical and hemodynamic outcomes of balloon aortic valvuloplasty in neonates and infants with critical aortic valve stenosis treated at a single pediatric cardiology center, with particular emphasis on procedural efficacy, early complications, and short-term clinical course.

## Materials and methods

### Study design and population

This retrospective single-center study included neonates and infants up to 12 months of age diagnosed with critical aortic valve stenosis who underwent balloon aortic valvuloplasty between 2016 and 2024 at a tertiary pediatric cardiology center.

Critical aortic valve stenosis was defined by severe left ventricular outflow tract obstruction associated with depressed systemic perfusion and/or duct-dependent systemic circulation. Patients with hypoplastic left heart syndrome or complex congenital heart disease requiring immediate surgical correction were excluded.

#### Institutional treatment strategy

At our institution, balloon aortic valvuloplasty (BAV) is considered the preferred first-line treatment for neonates and infants with critical aortic valve stenosis and a biventricular circulation. The decision to perform catheter-based intervention rather than primary surgical valvotomy was based on contemporary international practice, the less invasive nature of the procedure, and the potential for rapid hemodynamic stabilization.

Patients were considered candidates for BAV in the presence of severe left ventricular outflow tract obstruction associated with duct-dependent systemic circulation, impaired left ventricular systolic function, cardiogenic shock, or significant pressure gradients across the aortic valve. Surgical intervention was reserved for patients with unfavorable anatomy, failed catheter intervention, severe valve dysplasia not amenable to balloon dilation, or significant residual lesions requiring operative correction.

All treatment decisions were made by a multidisciplinary team including pediatric cardiologists, interventional cardiologists, cardiac surgeons, and intensivists.

### Pre-procedural assessment

All patients underwent comprehensive clinical and echocardiographic evaluation prior to intervention. Transthoracic echocardiography was used to assess:

- peak and mean transaortic pressure gradients;
- aortic valve morphology;
- left ventricular systolic function;
- presence and severity of aortic regurgitation.

Clinical status, including signs of low cardiac output, hemodynamic instability, and the need for prostaglandin E1 infusion, was documented.

The severity of aortic regurgitation was assessed using standard transthoracic Doppler echocardiographic criteria and categorized as none, mild, moderate, or severe according to contemporary pediatric echocardiographic recommendations.

### Balloon aortic valvuloplasty technique

Balloon aortic valvuloplasty was performed under general anesthesia using standard interventional techniques. Vascular access was obtained via the femoral artery. After crossing the aortic valve with a guidewire, balloon dilation was performed using a balloon sized according to the aortic annulus diameter. The balloon-to-annulus diameter ratio ranged between 0.8 and 0.9, consistent with established recommendations for neonatal and infant balloon aortic valvuloplasty. One or more balloon inflations were performed according to the immediate hemodynamic response and residual transvalvular gradient.

Balloon dilation was performed using Tyshak II balloon catheters (NuMED Inc., Hopkinton, NY, USA). In low-weight infants (< 2 kg), low-profile Tyshak Mini balloon catheters (NuMED Inc., Hopkinton, NY, USA) were used when appropriate. Coronary guidewires were utilized in selected cases with pinpoint aortic valve openings to facilitate crossing of the stenotic valve.

Procedural success was defined as a significant reduction in transaortic pressure gradient with acceptable aortic regurgitation and clinical stabilization.

### Post-procedural evaluation and follow-up

Echocardiographic assessment was repeated immediately after the procedure to evaluate residual gradient, left ventricular systolic function, and degree of aortic regurgitation. Early outcomes included in-hospital mortality, procedural complications, and the need for repeat intervention or surgical referral.

The duration of follow-up ranged from 15 days to 7.7 years. Long-term surveillance was performed using serial clinical and echocardiographic evaluations. Follow-up assessments focused on residual or recurrent aortic stenosis, progression of aortic regurgitation, left ventricular function, and the need for repeat intervention or surgical treatment.

### Statistical analysis

Continuous variables were tested for normality using the Shapiro–Wilk test. Normally distributed variables are presented as mean ± standard deviation (SD), whereas non-normally distributed variables are presented as median and interquartile range (IQR). Categorical variables are expressed as frequencies and percentages.

Paired comparisons between pre- and post-procedural hemodynamic parameters were performed using the paired Student’s t-test for normally distributed data and the Wilcoxon signed-rank test for non-normally distributed variables. A two-sided p-value < 0.05 was considered statistically significant.

Statistical analyses were performed using IBM SPSS Statistics version 26.0 (IBM Corp., Armonk, NY, USA).

## Results

Key hemodynamic and clinical outcomes are summarized in Table 1.

**Table 1:** Baseline Characteristics of the Study Cohort (*n* = 30)

Characteristic	Value
Number of patients	30
Age at intervention (days), mean ± SD	20.0 ± 14.3
Body weight (kg), mean ± SD	3.4 ± 1.5
Critical aortic stenosis	30 (100%)
Prostaglandin E1 infusion before intervention	30 (100%)
Peak systolic gradient (mmHg), mean ± SD	67.6 ± 19.0
Left ventricular ejection fraction (%), mean ± SD	48.2 ± 3.1
Aortic valve morphology	Predominantly bicuspid; unicuspid and tricuspid variants also observed
Balloon-to-annulus ratio	0.8–0.9
Early complications	16 (53.3%)
In-hospital mortality	4 (13.3%)
Reintervention during follow-up	5 (16.7%)
Repeat balloon aortic valvuloplasty	3 (10.0%)
Surgical referral during follow-up	2 (6.7%)

### Patient characteristics

A total of 30 neonates and infants with critical aortic valve stenosis underwent balloon aortic valvuloplasty during the study period. The cohort included both neonates and infants up to 12 months of age.

All patients required prostaglandin E1 infusion before intervention to maintain duct-dependent systemic circulation and adequate systemic perfusion. Prostaglandin E1 was administered at a dose of 0.05–0.1 µg/kg/min until the time of balloon aortic valvuloplasty.

Pre-procedural echocardiography demonstrated severe transaortic pressure gradients and varying degrees of left ventricular systolic dysfunction. Bicuspid aortic valve morphology represented the predominant anatomical subtype, consistent with the overall institutional experience, in which bicuspid morphology accounted for approximately three-quarters of patients with critical aortic valve stenosis. Unicuspid and tricuspid valve variants were less frequently observed. In addition, varying degrees of valve dysplasia and leaflet fibrosis were identified before intervention.

### Immediate hemodynamic outcomes

Balloon aortic valvuloplasty resulted in a marked immediate reduction in peak transaortic pressure gradients (Table 2), indicating effective relief of left ventricular outflow tract obstruction. The mean peak systolic gradient decreased by approximately 55%, reflecting a clinically meaningful hemodynamic improvement. Left ventricular systolic function improved in the majority of patients following intervention.

**Table 2:** Hemodynamic and clinical outcomes before and after balloon aortic valvuloplasty

Parameter	Before BAV	After BAV	*p*-value
Peak systolic gradient (mmHg)	67.6 ± 19	30.3 ± 3	< 0.05
Mean transaortic gradient (mmHg)	65.2 ± 2.7	30.3 ± 3.0	< 0.05
LV ejection fraction (%)	48.2 ± 3.1	62.3 ± 1.9	< 0.05
Mechanical ventilation (hours)	—	31 ± 12	—
ICU stay (days)	—	3 (range 2–40)	—
Reintervention during follow-up, n (%)	—	5 (16.7%)	—
Mortality, n (%)	—	4 (13.3%)	—
Early complications, n (%)	—	16 (53.3%)	—

Left ventricular systolic function improved or remained stable in most patients, as reflected by the increase in left ventricular ejection fraction (Table 2).

### Aortic regurgitation

New-onset or increased aortic regurgitation was observed following balloon dilation in a subset of patients, as detailed in Table 2. In most cases, regurgitation was mild to moderate in severity, while severe regurgitation remained uncommon across all follow-up time points (Table 3).

**Table 3:** Aortic Regurgitation Progression

Time point	None	Mild	Moderate	Severe
Before BAV	74%	26%	0%	0%
After BAV	30%	51%	19%	0%
12 months	25.5%	49%	25.5%	0%
36 months	32%	38%	30%	0%

### Procedural success and complications

Procedural success was achieved in the majority of patients. Balloon aortic valvuloplasty was generally well tolerated. No major vascular complications were observed. Early in-hospital mortality and complication rates are summarized in Table 4. Mortality remained low and was primarily related to the severity of underlying cardiac pathology rather than the procedure itself.

**Table 4:** Early Procedural and In-Hospital Complications

Complication	*n* (%)
Heart failure requiring intensive medical therapy	6 (20.0%)
Respiratory failure requiring prolonged ventilatory support	5 (16.7%)
Pulmonary atelectasis	3 (10.0%)
Bleeding complications	2 (6.7%)
Severe aortic regurgitation	0 (0%)
Major vascular complications	0 (0%)
Patients with at least one complication	16 (53.3%)

Some patients experienced more than one complication; therefore, individual categories may overlap

In addition to the complications listed above, transient hemodynamic instability and progression of aortic regurgitation were observed in selected patients during the early postoperative period. No sustained arrhythmias requiring invasive intervention were documented. No major vascular complications occurred.

Most complications were related to the severity of the underlying cardiac disease and perioperative clinical instability rather than technical aspects of the balloon valvuloplasty procedure. No major vascular complications, cardiac perforation, or procedure-related severe aortic regurgitation were observed.

#### Mortality analysis

Four patients (13.3%) died during the in-hospital period. Mortality was primarily associated with the severity of the underlying cardiac condition and pre-existing hemodynamic instability rather than direct procedural complications. All deaths occurred in critically ill patients presenting with severe left ventricular dysfunction, compromised systemic perfusion, and advanced heart failure at admission.

No deaths were directly attributable to vascular injury, cardiac perforation, device-related complications, or other technical aspects of the balloon valvuloplasty procedure. The observed mortality therefore reflects the high-risk nature of critical aortic valve stenosis in neonates and infants rather than procedural failure.

Given the retrospective design of the study and incomplete availability of detailed historical clinical records for all patients, a comprehensive patient-level analysis of mortality could not be performed.

### Early reintervention and surgical referral

During follow-up, repeat catheter-based intervention or referral for surgical treatment was considered in patients with significant residual aortic valve stenosis, recurrent obstruction, or progressive aortic regurgitation. The principal indications for further intervention included inadequate gradient reduction after the initial procedure and deterioration of valve function during follow-up.

Patients requiring further treatment were more likely to have complex valve morphology and impaired left ventricular function at baseline. Detailed information regarding balloon sizes used during repeat procedures and some procedural characteristics was not consistently available because of the retrospective nature of the study.

During follow-up, 5 patients (16.7%) required reintervention. Repeat balloon aortic valvuloplasty was performed in 3 patients (10.0%), whereas surgical intervention was required in 2 patients (6.7%).

Follow-up duration ranged from 15 days to 7.7 years, allowing assessment of early and intermediate-term clinical outcomes.

## Discussion

This study demonstrates that balloon aortic valvuloplasty provides effective early relief of left ventricular outflow tract obstruction in neonates and infants with critical aortic valve stenosis. The significant reduction in transaortic pressure gradients observed in our cohort is consistent with previously reported experiences and underscores the role of BAV as a valuable first-line intervention in early life.

From a pediatric perspective, timely relief of aortic obstruction is essential to restore systemic perfusion, support left ventricular recovery, and stabilize critically ill neonates. In our cohort, most patients exhibited early clinical improvement and stabilization following intervention, highlighting the practical clinical benefit of BAV beyond hemodynamic measurements alone.

Aortic regurgitation remains a recognized limitation of balloon aortic valvuloplasty. In the present study, regurgitation was predominantly mild to moderate, while severe regurgitation was uncommon. These findings align with existing literature and emphasize the importance of careful balloon sizing and procedural technique, particularly in patients with dysplastic valves.

The need for early reintervention or surgical referral in a subset of patients reflects the complex and progressive nature of critical aortic valve disease in infancy. In this context, balloon aortic valvuloplasty should be viewed not only as definitive therapy in selected cases but also as an effective bridging strategy allowing growth and clinical stabilization prior to surgery.

An important finding of this study is the substantial early reduction in transaortic pressure gradients accompanied by improvement in left ventricular systolic function. These results highlight the immediate physiological benefit of balloon aortic valvuloplasty, particularly in critically ill neonates with compromised systemic perfusion. The magnitude of gradient reduction observed in our cohort is comparable to previously published series, supporting the reproducibility of this technique across different clinical settings.

During follow-up, 16.7% of patients required repeat intervention or surgical treatment, which is consistent with previously reported pediatric BAV series.

Our findings are broadly consistent with data reported by the Congenital Heart Surgeons’ Society (CHSS) and the Valvuloplasty and Congenital Aortic Stenosis (VACA) registry. These large multicenter experiences demonstrated that balloon aortic valvuloplasty provides effective early relief of left ventricular outflow tract obstruction in neonates and infants, while highlighting the persistent risks of aortic regurgitation and the need for subsequent reintervention during long-term follow-up. The hemodynamic improvements observed in our cohort are comparable to those reported in these major registries, supporting the continued role of BAV as an important first-line treatment strategy in critical neonatal and infantile aortic stenosis.

Another clinically relevant observation is the relatively high incidence of mild-to-moderate aortic regurgitation following intervention. While this remains a known trade-off of the procedure, our data suggest that severe regurgitation is uncommon when appropriate balloon sizing strategies are applied. This emphasizes the importance of procedural expertise and careful patient selection in optimizing outcomes.

Importantly, balloon aortic valvuloplasty should not be viewed solely as a definitive intervention but rather as part of a broader treatment strategy. In many cases, it provides a crucial window for clinical stabilization and somatic growth, thereby improving the conditions for potential future surgical intervention.

### Limitations

The retrospective design and single-center nature of this study limit the generalizability of the findings. The relatively small sample size and focus on early outcomes preclude assessment of long-term valve function and reintervention rates. Nevertheless, the study provides valuable real-world data on the early management of critical aortic stenosis in a pediatric population. In addition, detailed patient-level information regarding ICU length-of-stay distribution, repeat intervention characteristics, balloon-to-annulus ratio distribution, and certain procedural variables was not consistently available in historical records because of the retrospective design of the study. Although key clinical and echocardiographic outcomes were available for all included patients, some detailed procedural data could not be reliably reconstructed from archived documentation.

Because of the retrospective nature of the study and incomplete availability of historical source documentation, detailed patient-level information regarding aortic annulus dimensions, balloon sizes used in individual procedures, associated syndromes, renal function parameters, and rapid pacing utilization could not be consistently retrieved for all patients. These variables were therefore not available for comprehensive statistical analysis.

## Conclusions

Balloon aortic valvuloplasty is a safe and effective initial treatment for critical aortic valve stenosis in neonates and infants, offering rapid hemodynamic improvement and acceptable early outcomes. The procedure plays an important role in the early stabilization of this high-risk population and may serve as definitive therapy or a bridge to subsequent surgical intervention.

## Data Availability

The datasets used and/or analyzed during the current study are available from the corresponding author on reasonable request.

## Abbreviations

BAV: Balloon aortic valvuloplasty
CAVS: Critical aortic valve stenosis
LV: Left ventricle
LVEF: Left ventricular ejection fraction
